# Supplementary material for: IFN-γ–producing self-reactive CD4+ T cells induce autoimmune adrenalitis in a mouse model of Addison’s disease
Source: JCI Insight. 2026 Aug 4;11(18):e201704. doi: 10.1172/jci.insight.201704 (PMC13596732; doi:10.1172/jci.insight.201704)
Supplement: Supplementary file 1 [file jciinsight-11-201704-s225.pdf]

Supplementary material: IFNG-producing self-reactive CD4$^+$ T cells induce autoimmune adrenalitis in a mouse model of Addison's disease

Arina Andreyeva[1], Juraj Michalik[1], Veronika Niederlova[1], Veronika Cimermanova[1,2], Ales Drobek[1], Radislav Sedlacek[3], Jan Prochazka[3], Juraj Labaj[3], Olha Fedosieieva[3], Waldemar Kanczkowski[4], Peter Draber[5], Andre Sulen[6] Ondrej Stepanek[#,1], Ales Neuwirth[#,1]

1 Laboratory of Adaptive Immunity, Institute of Molecular Genetics of the Czech Academy of Sciences, Prague, Czechia

2 Faculty of Science, Charles University, Prague, Czechia

3 Czech Centre for Phenogenomics & Laboratory of Transgenic Models of Diseases, Institute of Molecular Genetics of the Czech Academy of Sciences, Vestec, Czechia

4 Department of Internal Medicine III, University Hospital Carl Gustav Carus, Technische Universität Dresden, Dresden, Germany

5 Department of Immunobiology, University of Lausanne, Epalinges, Switzerland

6 Department of Clinical Science, Faculty of Medicine, University of Bergen, Norway

Correspondence should be addressed to Ondrej Stepanek, ondrej.stepanek@img.cas.cz and Ales Neuwirth, ales.neuwirth@img.cas.cz

Supplementary figure 1

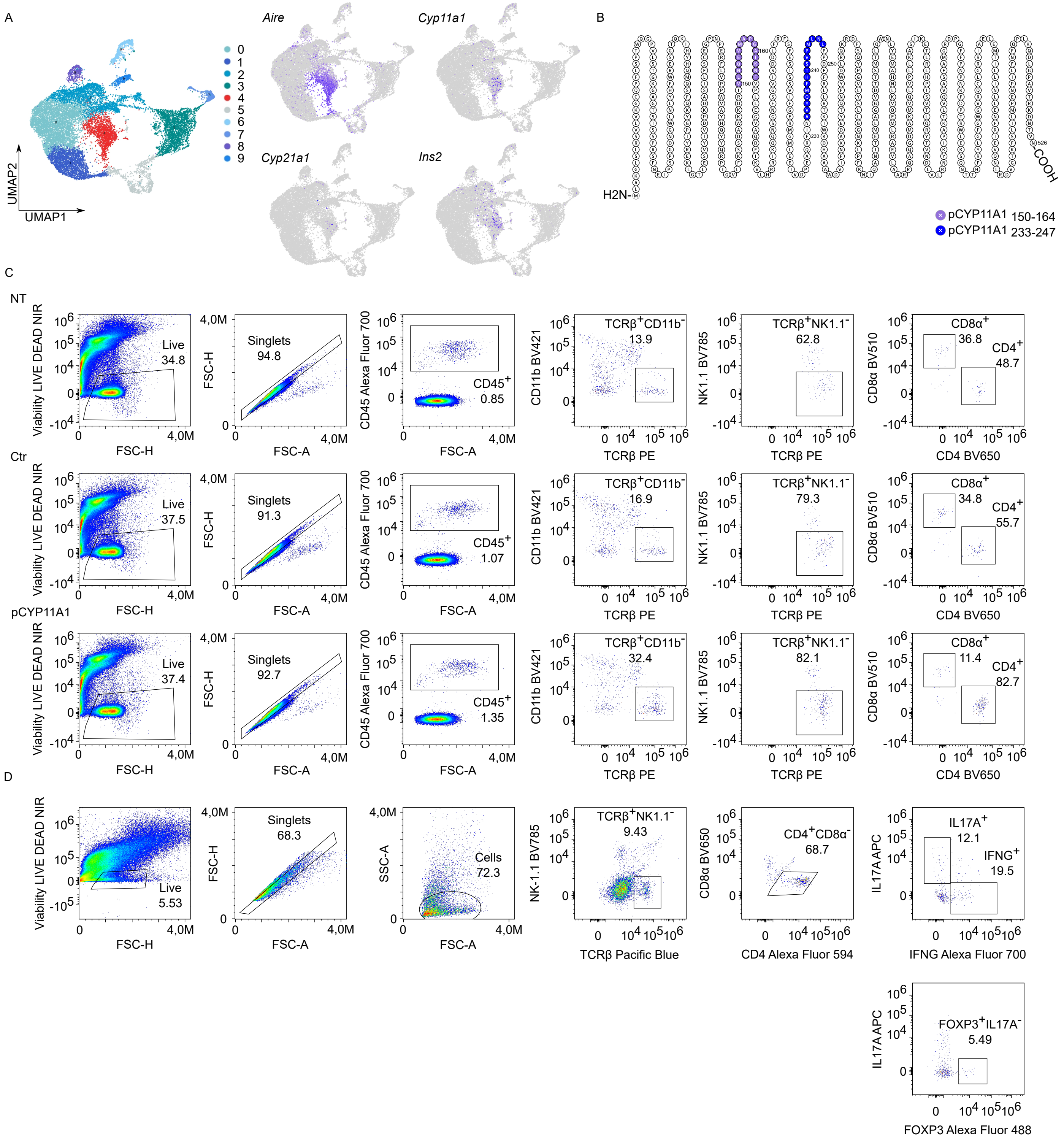

Supplementary Figure 1. ***Cyp11a1*** **expression in mTECs, immunogenic CYP11A1 peptides and identification of adrenal CD4$^+$ T cells.**

(A) UMAP of scRNA-seq data from TECs of 8-week-old female mice (also in Figure 1A) with feature plots displaying expressions of selected genes and highlighting Aire-expressing mTECs (cluster 4). Gene expression is visualized in shades of violet, with higher intensity reflecting greater expression levels.

(B) Visualization of the linear protein sequence of murine CYP11A1 (Protter software) (Omasits et al., 2014) with the two peptides used for immunization - CYP11A1$_{150\text{-}164}$ and CYP11A1$_{233\text{-}247}$ - highlighted in indicated colors. Peptides were selected based on predicted immunogenicity and MHC class II binding potential.

(C) Representative gating for identifying adrenal CD4$^+$ and CD8$^+$ T cells (Figure 1B).

(D) Representative gating for identifying Tregs and cytokine-producing CD4$^+$ T cells (Figure 1C).

Supplementary figure 2

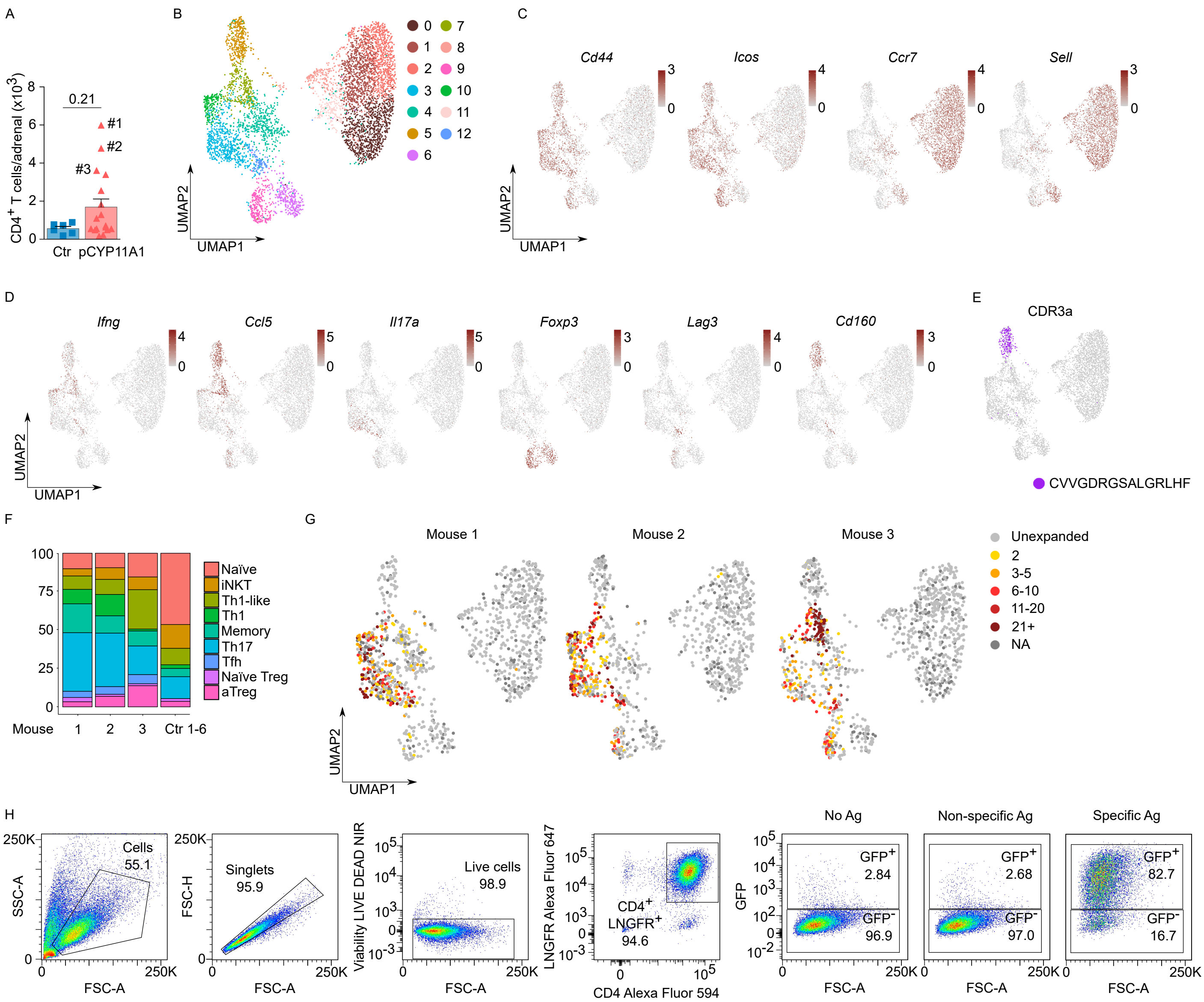

Supplementary Figure 2. Heterogeneity and antigen specificity of adrenal $CD4^+$ T cells

(A) Quantification of $CD4^+$ T-cell infiltration in adrenal glands of pCYP11A1-immunized and control mice at day 14 p.i. (n=6-17 mice/group).

Statistical significance was determined using two-tailed Mann-Whitney test; P values are indicated. Bars show the mean ± SEM; individual mice are shown as symbols.

(B) UMAP of scRNA-seq data showing 13 transcriptionally defined $CD4^+$ T cell clusters from adrenal glands and spleens. Five similar clusters with a naïve phenotype were merged and labeled in the final projection as "Naïve $CD4^+$" (Figure 1D).

(C) Expression of signature genes for naïve and effector $CD4^+$ T cell subsets in cells derived from adrenal glands and spleens.

(D) Expression of marker genes used to identify specific effector $CD4^+$ T cell subsets in cells derived from adrenal glands and spleens. In (C-D) color intensity correlates with the level of gene expression as indicated.

(E) Feature plot identifying the $CD4^+$ invariant NKT (iNKT) cell subset based on expression of the canonical CDR3α sequence (CVVGDRGSALGRLHF).

(F) Distribution of transcriptionally defined $CD4^+$ T-cell populations in adrenal glands only, shown for individual immunized mice (n = 3 mice) and pooled control mice (n = 6 mice).

(G) UMAP showing clonal expansion of $CD4^+$ T cells in adrenals and spleen from individual pCYP11A1-immunized mice. Color represents clone size, defined by the number of cells sharing the same TCR; "NA" denotes T cells without complete information about both TCR chains.

(H) Representative gating strategy for NFAT-GFP reporter T cell hybridoma (A5) clones used for antigen specificity assays (Figure 2G).

Supplementary figure 3

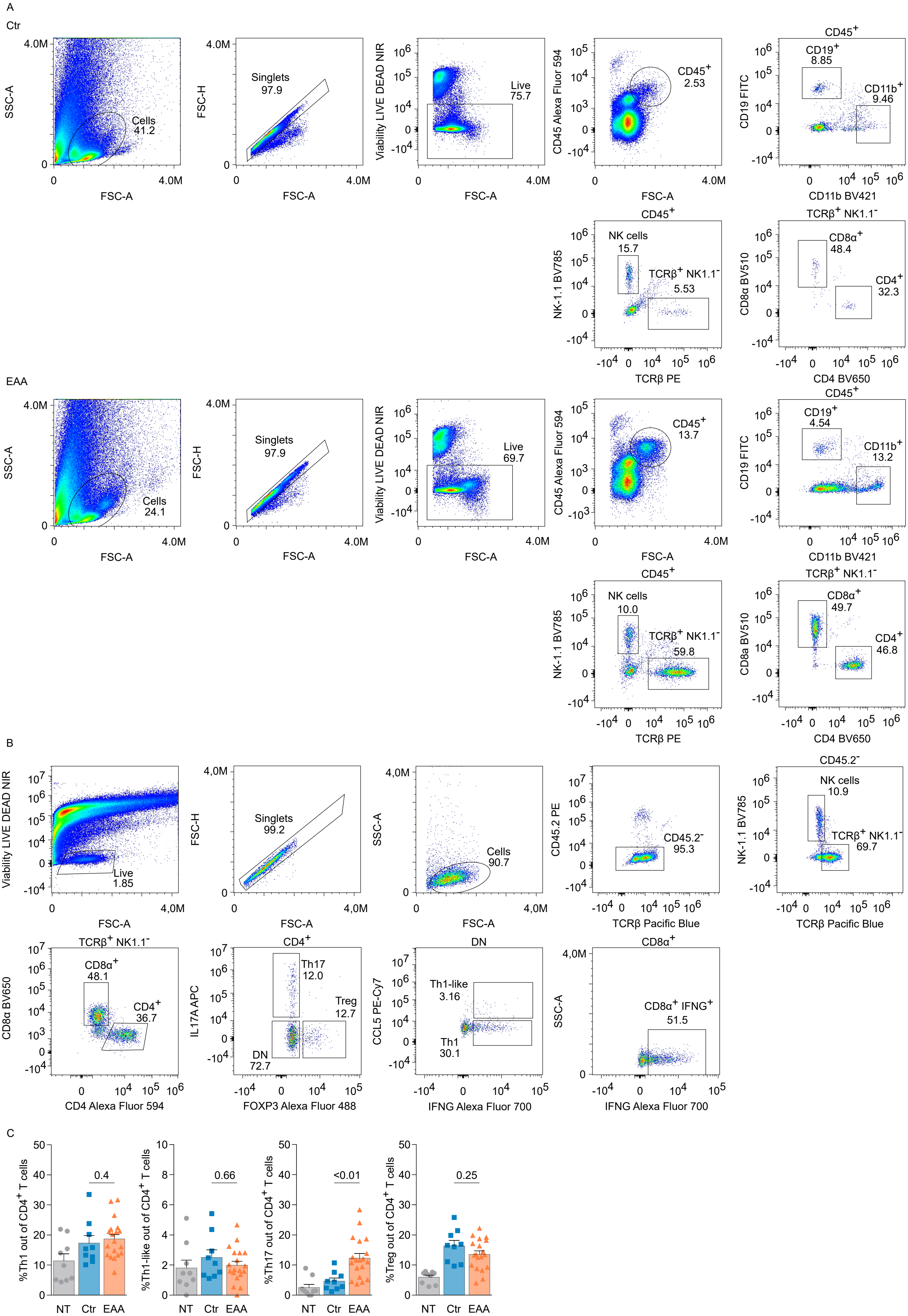

Supplementary Figure 3. Immune cell characterization in the adrenal glands in the EAA model.
(A) Representative gating for identifying major immune cell populations in adrenal glands of Ctr and EAA mice (Figure 3A).
(B) Representative gating for identifying $CD4^+$ effector T cell subsets (Figure 3C-D). CD45.2 PE antibody was injected 15 minutes prior to sacrifice, and blood-derived cells were excluded by gating on $CD45.2^-$ cells.
(C) Frequencies of $CD4^+$ effector T-cell subsets 3-4 weeks p.i., shown as percentages of total $CD4^+$ T cells (two independent experiments; n=9-19 mice/group).
Groups: non-treated (NT), control (Ctr), and EAA.
Statistical significance was determined using two-tailed Mann-Whitney test; P values are indicated. Bars show the mean ± SEM; individual mice are shown as symbols.

Supplementary figure 4

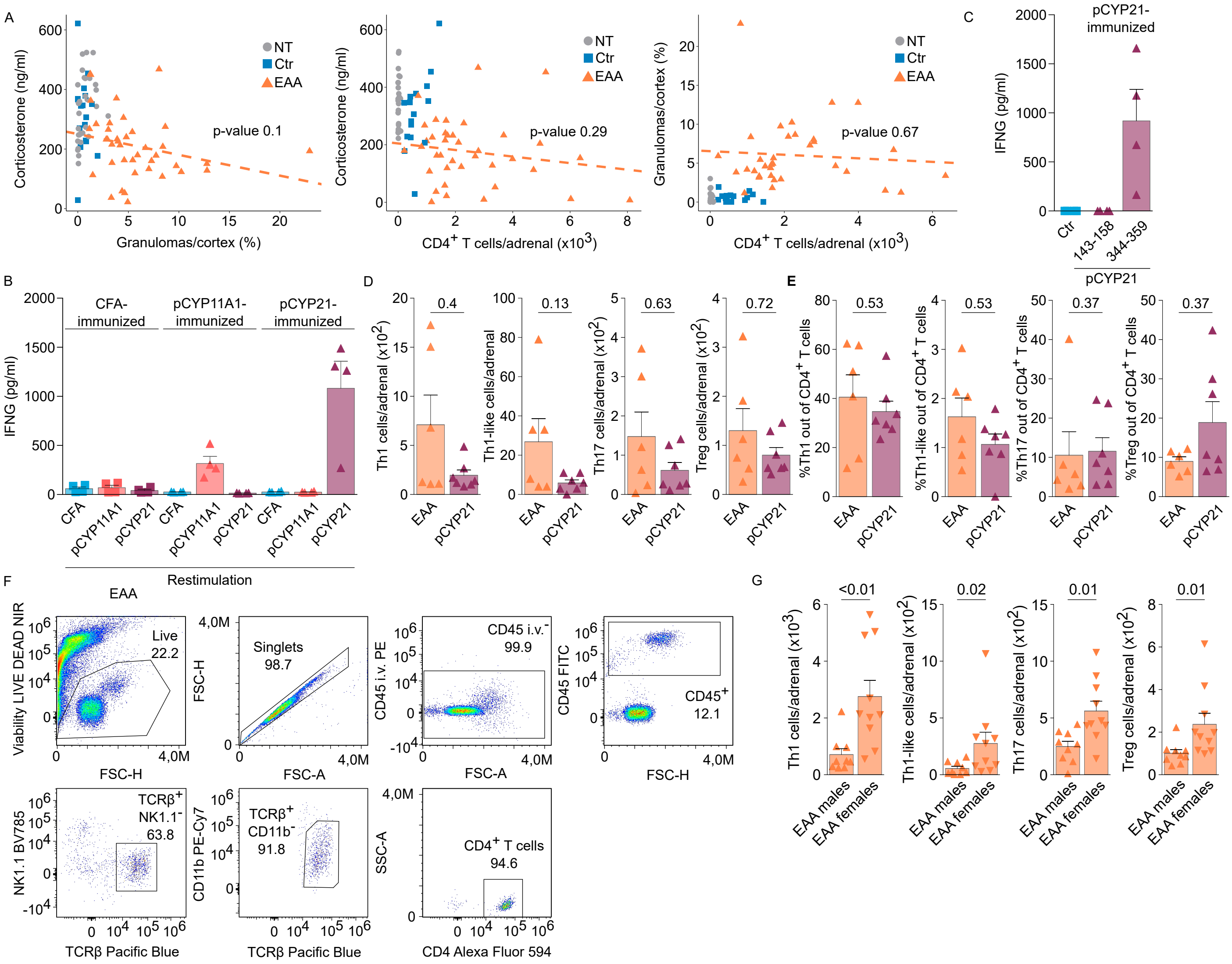

Supplementary Figure 4. Antigen-specific and sex-dependent features of adrenal pathology in the EAA model.

(A) Correlation between serum corticosterone levels, granuloma frequency, and $CD4^+$ T-cell infiltration in the adrenals of recipient mice (*Cd3e*$^{-/-}$, *Rag2*$^{-/-}$) in the adoptive EAA model 3 months p.i.

(B) IFNG levels in supernatants of splenocytes after restimulation with pCYP11A1 or pCYP21 ($CYP21_{143-158}$, $CYP21_{344-359}$) from previously pCYP11A1-or pCYP21-immunized B6 mice.

(C) IFNG levels in supernatants of splenocytes after restimulation with individual CYP21 peptides ($CYP21_{143-158,}$ $CYP21_{344-359}$).

(D) Absolute numbers and **(E)** frequencies of $CD4^+$ T-cell effector subsets infiltrating adrenal glands of *Cd3e*$^{-/-}$ recipients 3 months post-transfer of pCYP11A1 (EAA)-or pCYP21-restimulated $CD4^+$ T cells (two independent experiments; n=6-7 mice/group)

(F) Representative gating strategy for identification of $CD4^+$ T cells (Figure 6H). CD45.2 PE antibody was injected 15 minutes prior to sacrifice, and blood-derived cells were excluded by gating on $CD45.2^-$ cells.

(G) Absolute numbers of $CD4^+$ T-cell effector subsets infiltrating adrenal glands of *Rag2*$^{-/-}$ male and female recipients 3 months post-transfer (two independent experiments; n=9-10 mice/group). Cells were gated as in Figure S3D.

Omasits, U., C.H. Ahrens, S. Muller, and B. Wollscheid. 2014. Protter: interactive protein feature visualization and integration with experimental proteomic data. *Bioinformatics* 30:884-886.
